# E-MOTE: A Conceptual Framework for Emotion-Aware Teacher Training Integrating FACS, AI and VR

**DOI:** 10.3390/vision10010005

**Published:** 2026-01-19

**Authors:** Rosa Pia D’Acri, Francesco Demarco, Alessandro Soranzo

**Affiliations:** 1 Department of Cultures, Education and Society, University of Calabria, 87036 Cosenza, Italy; 2 Department of Physics, University of Calabria, 87036 Cosenza, Italy; francesco.demarco@unical.it (F.D.); alessandro.soranzo@unical.it (A.S.)

**Keywords:** teacher training, teacher noticing, social and emotional learning, facial action coding system, artificial intelligence, virtual reality, micro-expressions

## Abstract

This paper proposes E-MOTE (Emotion-aware Teacher Education Framework), an ethically grounded conceptual model aimed at enhancing teacher education through the integrated use of the Facial Action Coding System (FACS), Artificial Intelligence (AI), and Virtual Reality (VR). As a conceptual and design-oriented proposal, E-MOTE is presented as a structured blueprint for future development and empirical validation, not as an implemented or evaluated system. Grounded in neuroscientific and educational research, E-MOTE seeks to strengthen teachers’ emotional awareness, teacher noticing, and social–emotional learning competencies. Rather than reporting empirical findings, this article offers a theoretically structured framework and an operational blueprint for the design of emotion-aware teacher training environments, establishing a structured foundation for future empirical validation. E-MOTE articulates three core contributions: (1) it clarifies the multi-layered construct of emotion-aware teaching by distinguishing between emotion detection, perception, awareness, and regulation; (2) it proposes an integrated AI–FACS–VR architecture for real-time and post hoc feedback on teachers’ perceptual performance; and (3) it outlines a staged experimental blueprint for future empirical validation under ethically governed conditions. As a design-oriented proposal, E-MOTE provides a structured foundation for cultivating emotionally responsive pedagogy and inclusive classroom management, supporting the development of perceptual micro-skills in teacher practice. Its distinctive contribution lies in proposing a shift from predominantly macro-behavioral simulation toward the deliberate cultivation of perceptual micro-skills through FACS-informed analytics integrated with AI-driven simulations.

## 1. Introduction

Teaching is among the most emotionally complex and interpersonally demanding professions, requiring constant navigation of subtle affective cues and relational dynamics. Educators operate in environments where subtle emotional cues significantly influence student motivation, attention, and learning outcomes [1,2,3]. Developing emotional literacy and self-awareness, as emphasized in emotional intelligence–based approaches to education [4,5,6], is therefore central to teachers’ professional identity and classroom effectiveness. The ability to perceive and respond to students’ emotional states, especially micro-expressions lasting less than half a second, is a critical competency for emotionally responsive classroom management and inclusive pedagogy [7].

However, while emotions play a pivotal role in teaching effectiveness, teacher education programs rarely provide systematic, evidence-based training for perceiving and responding to subtle affective cues, particularly micro-expressions. Existing simulation platforms such as SimSchool focus primarily on macro-behavioral rehearsal and decision-making, but do not integrate real-time micro-expression analysis or affective feedback. Despite extensive research on Social and Emotional Learning (SEL) for students, teacher preparation programs still offer limited opportunities to practice real-time recognition and adaptive responses in safe, feedback-rich contexts [8,9]. This gap is mirrored in educational technology: while AI and VR show promise for teacher training, most systems remain limited to macro-behavioral rehearsal and provide little or no validated, real-time feedback on micro-expressions, which is essential for cultivating teachers’ perceptual precision [4,5,6,7,8,9,10].

Despite these advances, no existing teacher-training models systematically integrate validated facial coding, AI-driven affective feedback, and immersive simulation into a unified pedagogical framework.

To address this gap, this paper proposes the Emotion-Aware Teacher Education Framework (E-MOTE), a conceptual and design-oriented framework intended to integrate FACS, AI, and VR for emotion-aware teacher training. It is important to note that E-MOTE is presented as a theoretically structured blueprint for future development and validation, not as an empirically tested system. Against this background, E-MOTE is advanced as a conceptual response to the identified pedagogical and technological gaps in current teacher education models. The intended primary audience includes teacher educators, instructional designers, and researchers in educational technology and affective computing, while remaining highly relevant to practicing teachers and school leaders interested in emotion-aware pedagogy.

This study is structured around three core questions that articulate its rationale and intended contribution.

First, why is emotional perception training essential for teachers, and why do current preparation models overlook this critical skill?

Second, how might this gap be addressed through the integration of the Facial Action Coding System (FACS) [11,12], Artificial Intelligence (AI), and Virtual Reality (VR) within a unified framework for emotion-aware teacher training?

Third, what conceptual and operational framework is proposed in this paper to systematically integrate FACS, AI, and VR for emotion-aware teacher education, and how are its core components and training processes theoretically articulated?

Empirical evidence further confirms that teachers often struggle to accurately recognize students’ emotional states, which can negatively affect classroom climate and learning outcomes.

For instance, studies [13,14] reported low levels of accuracy in teachers’ judgments of students’ well-being and frequent misinterpretations of children’s facial expressions.

In doing so, E-MOTE extends existing simulation-based approaches by treating emotion perception itself, rather than classroom management alone, as a trainable professional competency. While previous models focus on classroom management or general emotional competencies, they neglect the fine-grained perceptual processes involved in micro-expression recognition and fail to provide an integrated pipeline combining validated facial coding systems, AI analytics, and immersive simulations.

Technological components.

Within this framework, E-MOTE integrates three technological components:

- FACS (Facial Action Coding System) for scientifically established decoding of subtle facial cues;
- Artificial intelligence (AI) for automated analysis and adaptive, real-time feedback;
- Virtual reality (VR) for immersive, controlled environments in which teachers can safely experiment with emotionally complex classroom situations.

Theoretical and methodological foundations.

Conceptually, E-MOTE builds on three complementary foundations:

1. The ability-based model of emotional intelligence [6];
2. Design-based research (DBR) methodologies [15];
3. Simulation-based teacher-training environments such as SimSchool [16,17].

E-MOTE is presented as a research-informed conceptual framework that bridges interdisciplinary theory and educational practice through a clearly defined structural model and remains open to iterative refinement and future empirical validation. In addition, E-MOTE conceptualizes emotion-aware teaching through four interconnected levels—emotion detection, emotion perception, emotion awareness, and emotion regulation—distinguishing computational inference from human perceptual and reflective competencies.

Table 1 provides a terminological clarification of these four levels, specifying their domains and pedagogical focus.

These four interconnected levels structure the emotional architecture of E-MOTE, linking automated detection to human perception, reflective awareness, and pedagogical regulation. The following section situates this multi-layered model within the broader literature on emotion-aware learning environments.

## 2. Related Research and Gap Identification

Although interest in technologies supporting emotion inference in education is growing, the current scholarly landscape remains fragmented and siloed. Meta-analytic evidence demonstrates that simulation-based learning can significantly support higher-order cognitive and professional skills in higher education, particularly when simulations are structured around feedback, reflection, and iterative practice [18]. Most contributions investigate emotion inference separately from artificial intelligence (AI) and virtual reality (VR), with only a few attempts at systematic integration. Well-established simulation platforms like SimSchool have advanced scenario-based teacher training, yet they do not incorporate validated micro-expression decoding or real-time affective feedback. Bibliometric and visual analyses confirm this fragmented landscape, showing both rapid growth and persistent silos in research on AI in education.

To strengthen the contemporaneity and comparative scope of this discussion, the following review highlights representative recent studies that bridge AI, affective computing, and immersive learning. Lampropoulos et al. [19] present a bibliometric review showing the accelerating convergence between affective computing and immersive (AR/VR) learning, underscoring the need for emotion-aware simulation tools. Tan et al. [20] synthesize the emerging role of AI in teacher professional development and classroom practice, emphasizing the importance of human-centered design and ethical oversight in educational AI systems. Recent advances in generative AI, including large language models (LLMs), have further expanded the educational AI landscape by enabling adaptive content generation, automated feedback, and conversational tutoring. However, integrating these technologies into teacher education raises multifaceted socio-technical and ethical issues related to transparency, data privacy, and pedagogical dependency [21,22].

Han [23] offers a systematic review of VR in teacher education, identifying growing evidence for immersive/mixed-reality training while calling for stronger methodological reporting. At the teacher–AI interface, Feldman-Maggor et al. [24] show that explainable-AI (XAI) features can enhance teachers’ trust and acceptance of AI-enhanced educational systems. Explainable Artificial Intelligence (XAI) refers to a set of methods and design principles aimed at making AI decision-making processes transparent, interpretable, and understandable to human users, particularly in high-stakes domains such as education [24].

Complementary studies on classroom-based facial expression recognition [25] and meta-analyses on VR’s impact on engagement [26] further demonstrate the technical feasibility and pedagogical promise of these approaches.

Together, these works highlight the need for an integrated and ethically grounded framework capable of uniting these technological strands into a coherent pedagogical model. AI-based emotion inference has been applied in educational contexts for emotion inference, learning analytics, and adaptive instruction [27,28]. Luckin and colleagues [28,29] argue that AI should be designed not to replace human intelligence but to augment it, fostering the development of ethical, emotional, and cognitive skills in teachers and learners. Consistent with this perspective, previous studies [30,31,32] demonstrate AI’s potential to personalize learning based on students’ affective states.

VR shows strong potential for cultivating teachers’ socio-emotional competencies through high-fidelity simulations. Recent research [33] demonstrates VR’s effectiveness in simulating complex classroom dynamics that foster empathy, classroom management and reflective capacity. Yet current VR applications seldom embed real-time emotion inference or dynamic AI-driven feedback, limiting their contribution to training teachers’ perceptual micro-skills for decoding subtle expressions during live instruction.

Recent efforts have begun to integrate AI-driven affective feedback mechanisms into VR-based teacher-training systems. For instance, Zhang et al. [34] explore hybrid environments where teachers interact with AI-powered avatars capable of emotional responsiveness. While promising, these models typically do not incorporate validated decoding systems like FACS and overlook systematic micro-expression analysis.

Well-established simulation-based platforms such as SimSchool, TeachLivE and ClassSim have significantly contributed to the evolution of scenario-based teacher training, offering structured environments for decision-making and classroom management practice [35,36]. These systems represent important advances in immersive pedagogy; however, current peer-reviewed literature does not report the integration of validated micro-expression decoding systems or real-time affective feedback grounded in FACS-based analysis within these platforms. Within this landscape, E-MOTE positions itself as a complementary conceptual framework aimed at extending existing simulation paradigms toward perceptual and emotion-aware teacher training.

To the best of our knowledge, no systematically documented models currently attempt to integrate these elements into a coherent and ethically grounded framework, and existing approaches remain partial or limited in scope. The proposed E-MOTE framework explicitly employs FACS to decode emotional micro-expressions, embeds AI-powered real-time feedback into VR training scenarios, aligns implementation with ethical standards such as the General Data Protection Regulation (GDPR; Regulation (EU) 2016/679) [37] and articulates pedagogical pathways for professional development and classroom application. Ethical, bias, and privacy considerations are further detailed in the Limitations section.

Table 2 summarizes representative studies on AI- and VR-based approaches to teacher training (2022–2024), highlighting the distribution of key design features and making visible persistent gaps in validated emotion-decoding and ethical safeguards.

The comparison is strictly conceptual and theory-driven, and does not imply any claims regarding empirical effectiveness, implementation maturity, or performance differences among the systems considered. Its purpose is to highlight how specific design features are distributed across current approaches and to contextualize the positioning of E-MOTE within the existing design landscape, making visible persistent gaps in validated emotion-decoding and ethical safeguards (see Limitations). This comparative overview does not aim to benchmark E-MOTE against existing systems in terms of performance or effectiveness, but rather to situate it conceptually within the evolving landscape of AI- and VR-based approaches to teacher education.

For this analysis, “pedagogical integration” is assessed based on the explicit and central connection between the technological tool and a defined pedagogical theory or a structured pathway for professional development. A rating of ‘✔’ (present) indicates that the study explicitly grounds its application in educational theory (e.g., experiential learning, reflective practice) and outlines how the technology leads to the development of specific, transferable teaching competencies. A rating of ‘Partial’ indicates that while educational benefits are mentioned, the paper lacks a clear, methodological link to a pedagogical framework or a detailed model for how the tool fosters professional growth.

For example, Chiu et al. [36] emphasize AI’s potential to personalize learning, yet their systems remain focused primarily on student modeling rather than teacher development. Rodríguez-Andrés et al. [32] show the effectiveness of VR for simulating classroom dynamics but without AI-driven feedback. Taken together, these studies highlight both the promise of AI-VR integration and the persistent absence of robust and ethically grounded approaches to micro-expression analysis.

Unlike existing VR-based simulation platforms for teacher education, which typically rely on scripted interactions and macro-behavioral feedback, E-MOTE introduces a theoretically grounded and ethically oriented integration of the Facial Action Coding System (FACS), Artificial Intelligence (AI), and Virtual Reality (VR) specifically designed to enhance teachers’ perceptual micro-skills.

Current systems mainly focus on classroom management and decision-making within pre-programmed scenarios, providing only limited analytical access to the subtle affective data that underpin authentic teacher–student attunement.

E-MOTE addresses these limitations by proposing the integration of FACS within an AI-driven analytic engine capable of decoding involuntary micro-expressions and delivering adaptive, real-time feedback through immersive VR simulations. Empirical studies suggest that systematic training in micro-expression recognition may improve teachers’ ability to interpret and respond to students’ emotions, fostering more supportive and emotionally balanced classroom environments [13,14,37,38].

Through this human-centered and evidence-informed approach, E-MOTE reframes simulation-based teacher training from behavioral rehearsal to perceptual learning, bridging the gap between conceptual awareness and embodied perception and regulation of emotions in authentic classroom contexts.

By incorporating explainable artificial intelligence (XAI) techniques and systematic bias-auditing protocols, E-MOTE is designed to promote transparency, fairness, and pedagogical trustworthiness—dimensions often overlooked in previous approaches.

However, most existing systems still lack actionable feedback and rarely utilize validated tools such as FACS. These models rarely include transparent ethical safeguards or mechanisms for algorithmic accountability. The proposed framework addresses these critical gaps by combining scientifically validated micro-expression decoding (FACS), real-time adaptive feedback (AI), and immersive, ethically governed VR simulations into a unified and transparent ecosystem. This integration not only bridges methodological silos across affective computing and teacher education but also introduces an ethical and explainable AI layer that ensures interpretability and fairness.

The framework therefore aims to delineate a coherent conceptual and technological positioning within the current research landscape, positioning itself as an integrative model that unites validated emotion-decoding methods, adaptive simulations, and ethical governance within a scalable ecosystem for teacher education.

The rest of this paper is structured as follows. After the Introduction (Section 1), Section 2 reviews the related literature to contextualize and identify the research gap addressed in Section Research Gap. Section 3 presents Preliminaries on FACS as the methodological foundation. Section 4 introduces the proposed model (E-MOTE), detailing its structural layers and operational workflow (Section 4.1), advanced use cases (Section 4.2), and core professional competencies and ethical foundations (Section 4.3). Section 5 outlines experimental validation strategy. Section 6 provides the Discussion, Section 7 presents Limitations, and Section 8 concludes the paper.

Taken together, the reviewed evidence (and the comparative mapping in Table 2) indicates a persistent research gap in integrated, ethically governed AI–FACS–VR training models.

### Research Gap

Despite growing interest in AI and VR for education, current teacher training programs still offer limited structured and systematically validated opportunities to practice emotional perception, particularly with regard to subtle and involuntary emotional cues such as micro-expressions. Existing systems tend to prioritize macro-behavioral responses or scripted interactions and do not report the use of scientifically validated methods such as FACS-based micro-expression decoding or the integration of explainable AI-driven affective feedback mechanisms. As a result, the development of teachers’ perceptual competencies for emotionally responsive and inclusive pedagogy remains insufficiently supported within prevailing training models.

The E-MOTE framework is designed to address this gap by proposing a structured, theory-informed approach to emotion-aware teacher education. This conceptual gap reveals a structural deficiency in prevailing teacher-training paradigms, particularly regarding the development of perceptual-emotional competencies.

## 3. Preliminaries: FACS as a Methodological Foundation

This section introduces the Facial Action Coding System (FACS) as the methodological foundation for the perceptual component of the E-MOTE framework.

The FACS is a standardized and scientifically validated methodology for objectively coding brief, involuntary facial muscle movements (micro-expressions) that may signal underlying affective states during teacher–student interactions. These micro-expressions, lasting between 1/25 and 1/4 of a second [7,8,9,10,11,12,13,14,15,16,17,18,19,20,21,22,23,24,25,26,27,28,29,30,31,32,33,34,35,36,37,38] are critical in educational settings, as students may attempt to conceal feelings of confusion, frustration, or anxiety. FACS enables the identification of specific facial muscle movements, termed Action Units (AUs), each corresponding to a specific facial muscle activation pattern that may be associated with affective processes marker that can be systematically observed and interpreted. For educators, FACS training sharpens perceptual accuracy, shifting from vague impressions of student affect to more finely grained and pedagogically actionable cues.

Research has demonstrated that with proper training, observers can significantly improve their ability to detect and interpret these subtle facial cues [39,40]. For a teacher, this skill is not about becoming a clinical diagnostician, but about gaining a more accurate and timely understanding of the classroom climate and individual student needs.

Table 3 summarizes the Facial Action Units (AUs) most relevant to emotion-related facial movements, together with their functional description.

It is crucial to note that emotions are typically expressed through the co-occurrence of multiple AUs rather than single markers. For instance:

- Sadness often involves AU1, AU4, and AU15 (lip corner depressor), signaling a need for empathetic connection or support.
- Genuine happiness or engagement is reflected in the concurrent activation of AU6 (cheek raiser) and AU12 (lip corner puller), the “Duchenne smile,” which can serve as positive feedback for a teacher’s instructional approach.
- Boredom or disengagement is often signaled by AU17 (chin raiser) and AU25 (lips part), indicating a need to increase the lesson’s pace, interactivity, or relevance.

By learning to recognize these nuanced AU combinations, educators can more accurately interpret students’ emotional states and respond with timely, supportive actions, such as intervening before frustration escalates or identifying confusion masked by silence. This perceptual skill is inherently contextual and holistic, emerging from the interplay between local facial cues and global perceptual organization shaped by neural and sociocultural factors [41,42,43].

### 3.1. Epistemological and Contextual Considerations of FACS in Education

While FACS provides a standardized method for coding facial movements, its application in educational contexts requires careful epistemological scrutiny. FACS is rooted in the basic emotion theory, which has been critiqued for its Western bias and limited generalizability across cultures. Cross-cultural studies show that identical facial configurations can be interpreted differently depending on cultural norms, display rules, and contextual cues. In classrooms, where student-teacher relationships, developmental stages, and individual differences shape emotional expression, FACS-based coding should not be treated as a deterministic mapping to internal emotional states. Instead, within the E-MOTE framework, FACS serves as a training scaffold to heighten teachers’ perceptual acuity, not as a diagnostic tool. Teacher interpretation remains central, guided by cultural responsiveness, contextual awareness, and ethical reflection.

### 3.2. Contextual Interpretation and Culturally Responsive Application

Neuroaesthetic studies, including investigations into the expressive ambiguity of Leonardo’s portraits, highlight how facial meaning emerges from dynamic perceptual integration rather than fixed facial markers, reinforcing the relevance of a holistic interpretative approach in educational contexts [44,45,46,47]. This observation aligns with neuroscientific evidence highlighting the integrated nature of perceptual and emotional processing in the brain [14].

Furthermore, cross-cultural research shows that identical facial configurations can elicit divergent interpretations, revealing differences in recognition thresholds and affective meanings [34,35,36,37,38,39,40,41,42,43,44,45,46,47,48]. Recent empirical evidence further supports these findings [49,50], showing systematic cultural variations in emotion inference accuracy and intensity perception.

This variability underscores the importance of interpreting facial cues through culturally responsive lenses [51,52], a principle that is central to the adaptable design of the E-MOTE framework. This understanding also aligns with the open-world perspective adopted by E-MOTE, which acknowledges that emotion inference is inherently multi-factorial and context-dependent. Rather than seeking deterministic classification, the framework promotes reflective, situational interpretation of affective cues—consistent with human-centered AI principles and culturally responsive pedagogy.

## 4. The Proposed Model: E-MOTE

Teacher education programs often lack structured opportunities for trainees to practice recognizing subtle, real-time emotional cues in safe yet realistic settings. As a result, emotional perception, teacher noticing, and regulation skills are not yet systematically supported at the point of classroom entry. The E-MOTE framework addresses this pedagogical gap by combining FACS-based micro-expression analysis, explainable AI inference, and adaptive VR feedback, proposing an integrated conceptual architecture that links perceptual input, pedagogical response, and reflective insight.

While E-MOTE builds on the AI–FACS–VR triad, the framework is operationally articulated into four functional modules, with reflective analytics constituting a distinct pedagogical layer rather than a technological component. All descriptions of components, workflows, and interactions in this section are presented for conceptual clarity and design logic, not as specifications of an implemented system.

**Figure 1:**
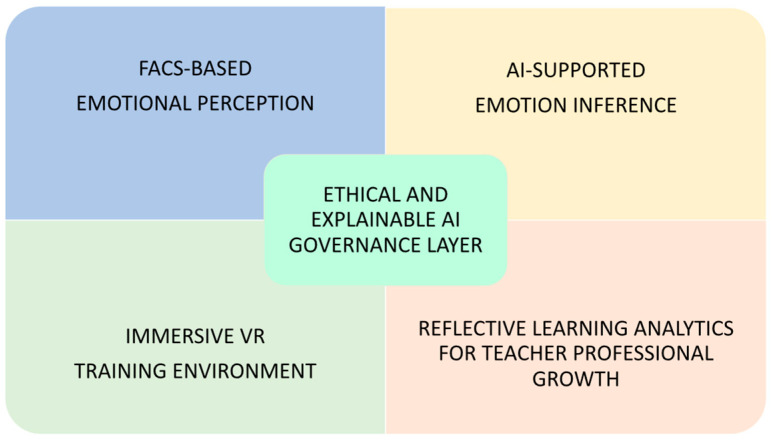
Conceptual architecture of the E-MOTE framework.

Within E-MOTE, FACS operates at two complementary levels: as a computational decoding standard embedded in the system architecture (Figure 1) and as a perceptual training reference supporting teachers’ professional noticing. Affective computing, defined as the use of computational systems to recognize, interpret, and respond to emotions [53], provides the interdisciplinary backbone of this integration. Advances in combining FACS with computer vision and deep learning architectures enable increasingly accurate decoding of facial muscle activations [26,54,55,56], while recent studies highlight the pedagogical and technical benefits of integrating VR with affective computing [18,19,20,21,22,23,24,25,30].

Taken together, these components position E-MOTE as a culturally responsive and potentially scalable pedagogical model for both pre-service and in-service teacher education. The overall conceptual architecture of the E-MOTE framework is illustrated in Figure 1, while the structural layers and operational logic of the model are detailed in Section 4.1, which constitutes the core contribution of this work.

Figure 1 illustrates the core conceptual components of the E-MOTE framework and their functional relationships. It represents a non-procedural, research-informed architecture rather than a sequential or fully implemented system.

As shown in Figure 1, E-MOTE is articulated as a conceptual architecture in which perceptual, interpretative, pedagogical, and reflective dimensions are functionally related rather than arranged in a fixed operational sequence. FACS-based micro-expression detection, AI-supported emotion inference, immersive VR-based feedback, and reflective learning analytics are conceptualized as interconnected components supporting emotion-aware teacher training. The reflective analytics dashboard aggregates post-session emotional dynamics to support metacognitive development, while adaptive feedback is delivered through real-time in-simulation VR cues and post-session reflective analytics.

Several technical and ethical challenges underpin this architecture. In particular, the translation of facial expressions from the physical to the virtual domain requires advanced animation techniques capable of mapping FACS-defined facial landmarks onto fully rigged 3D models, raising issues of data fidelity, privacy, and biometric governance—especially when minors are involved. These constraints directly inform Phase 1 of the E-MOTE validation roadmap, where dataset selection, bias calibration, consent pipelines, and secure biometric governance are treated as primary feasibility conditions rather than ancillary safeguards.

Although sequential in execution for explanatory purposes, all modules operate under a common ethical and explainable-AI layer that functions as a transversal governance mechanism rather than a discrete procedural phase. The operational workflow of these components is detailed in Section 4.1.

### 4.1. Structural Layers and Operational Workflow of the E-MOTE Framework

This section describes the structural layers and operational workflow of the core AI–FACS–VR component of E-MOTE, illustrating how raw facial and affect-related data captured during VR simulations are conceptually processed and transformed into pedagogically meaningful feedback for teacher-trainees. The workflow is described in discrete steps for explanatory purposes, but it represents a conceptual integration model rather than a technically implemented pipeline.

The following components are described separately for analytical clarity. Conceptually, however, E-MOTE operates as an interconnected and non-linear framework, as illustrated in Figure 1, rather than as a sequential or executable workflow.

Step 0—Ethical and privacy safeguards (see also Limitations):

Before any data processing occurs, E-MOTE requires explicit ethical safeguards, including informed consent, data minimization, pseudonymization, and bias audits, in compliance with GDPR [37] and UNESCO recommendations [57].

In accordance with the European Commission’s Ethical Guidelines for Trustworthy AI [20], the framework embeds an ethical-by-design approach that promotes transparency, fairness, and human oversight. These principles are operationalized through explainable AI (XAI) mechanisms [24], which enable users to inspect how specific Action Units (AUs) and contextual variables contribute to affective inferences.

Interpretable dashboards are designed to display fairness metrics and bias heatmaps, while local data processing prevents the export of raw video beyond the simulation environment. Although presented as Step 0 for explanatory clarity, ethical and explainable-AI safeguards operate as a transversal governance layer regulating all subsequent stages rather than as a discrete procedural phase.

Step 1—Data acquisition:

Within the VR environment, virtual student avatars are designed with realistic, FACS-compliant facial rigging. The system is conceptualized as generating and registering corresponding AU configurations for these avatars based on pre-programmed emotional responses to the teacher-trainee’s actions.

Step 2—Identification of AUs:

Computer vision algorithms, such as Convolutional Neural Networks (CNNs) trained on annotated datasets (CK+ [56]) or modern vision models integrated in Large Language Models (LLM), are envisioned as analyzing facial configurations in real time to estimate the activation of AUs within the simulated interaction.

Step 3—Processing and interpretation of affective patterns:

The system maps identified AU patterns to hypothesized affective states (AU1+AU4 → sadness/worry; AU6+AU12 → engagement), with deep neural networks supporting context-sensitive interpretation of these patterns within the classroom scenario.

Step 4—Real-time pedagogical feedback:

This step represents the pedagogical core of the framework, where affective insights are translated into reflective training support. Feedback is envisioned in two non-intrusive forms: in-simulation cue: a subtle, color-coded halo or icon appears above the avatar’s head (amber for “frustration” indicated by AU4, blue for “confusion” indicated by AU1). This allows the teacher to practice recognizing cues and adjusting their approach during the interaction.

Post-simulation debriefing dashboard: after the scenario, the teacher reviews a timestamped log of the emotional states they encountered, paired with video clips of their interactions. This dashboard highlights moments where key micro-expressions were present and allows for reflection on their responses, directly fostering the “reflective growth” competency.

This workflow conceptualizes the core of E-MOTE’s proposed training process, transforming VR simulations into reflective learning experiences. Empirical evidence supports the pedagogical rationale of such approaches, as immersive VR environments have been shown to enhance presence, emotional engagement, and reflective processing [58].

### 4.2. Advanced Use Cases of the AI-FACS–VR Integration

Building on the operational workflow, this subsection presents advanced pedagogical use cases, illustrating how the framework could be applied to complex and authentic teacher-education scenarios.

- Practicing inclusive classroom management: VR enables the creation of highly realistic simulations capable of replicating the multilayered dynamics and challenges of diverse classroom environments.
- Scenarios may include mediating interpersonal conflicts or de-escalating a frustrated student (practicing responses to AU4 and AU7 patterns commonly associated with heightened tension or frustration). These immersive experiences foster reflective awareness by allowing teachers to observe and adapt their behavioral responses during emotionally charged situations.
- Pedagogical transfer: the proposed feedback mechanisms are intended to support teachers in calibrating their ability to detect early signs of disengagement (AU17) or anxiety (AU1+AU20), enabling them to make timely instructional decisions like reframing a task or offering validation before a student fully disengages.
- Developing culturally responsive cue recognition: simulations can be designed to model culturally specific nuances in emotional expression, training teachers to avoid misinterpretations rooted in cultural bias. The AI feedback can be calibrated to different cultural datasets, making the teacher aware that the intensity or meaning of an AU configuration (like AU12 for a smile) can vary.
- Formative assessment of teacher noticing: the framework is designed to provide aggregated, structured indicators on a teacher’s “noticing” skills. For instance, a post-session report might indicate that the teacher consistently overlooked subtle markers of confusion (AU1) in a specific student avatar, identifying a concrete area for targeted growth.

In this context, the synergistic use of AI and VR provides an advanced technical-pedagogical solution for simulating and analyzing complex educational scenarios [22]. These scenarios cultivate metacognitive skills and support self-regulated learning—both essential for developing transferable, context-sensitive teaching competencies.

Such immersive scenarios allow teachers to practice real-time emotional perception in safe, dynamic environments. This integration conceptually bridges the gap between knowing about emotions and expertly perceiving them in the fast-paced, ever-changing reality of classroom interaction.

#### Technical Integration and Cost Considerations

From a technical perspective, the proposed integration of VR and AI follows a modular, cost-aware architecture designed for educational feasibility. The VR component is envisaged to be built on widely accessible game engines (Unity) to ensure compatibility with consumer-grade standalone headsets, balancing visual fidelity with performance on constrained hardware. AI processing—particularly for facial action unit detection—is designed for on-device inference where possible, while more computationally intensive models (for reflective feedback) would be handled via local external units to avoid cloud dependency. This localized approach supports both cost containment and compliance with data protection regulations, a critical concern when processing sensitive affective data from minors. These technical choices reflect a deliberate design stance prioritizing accessibility, privacy, and scalability within real-world educational budgets.

### 4.3. Core Professional Competencies and Ethical Foundations

Building on the technical workflow described above, this section introduces the professional and ethical competencies that the E-MOTE framework aims to develop, providing the conceptual foundation for the pedagogical discussion that follows. Moving beyond the technological integration of AI and VR outlined previously, this section focuses on the four core teaching competencies cultivated through AI–FACS feedback and immersive VR simulations, all grounded in robust ethical and pedagogical principles. They are:

- Emotional attunement: the ability to detect and interpret students’ facial cues with increasing perceptual accuracy, including micro-expressions, in conjunction with AI-supported detection systems, and to integrate this information to adapt communicative tone and teaching strategies in real time [59]. This aligns with the ability model of emotional intelligence, which defines EI as a set of cognitive-emotional skills including perception, understanding, and regulation of emotions [5,6].
- Empathic responsiveness: practiced through immersive VR scenarios that allow teachers to simulate context-sensitive reactions and emotionally supportive behaviors. This process is supported by evidence that emotionally engaging and cognitively structured learning experiences enhance both pedagogical effectiveness and learner outcomes [60,61], as well as presence-related research showing that immersion fosters psychological engagement in VR environments [62].
- Reflective growth: developed through self-assessment dashboards and post-simulation analytics that translate micro-expression data into tailored professional development goals. This aligns with a view of teacher learning as inquiry-based and embedded within communities of practice [63]. Reflective growth is supported through the reflective analytics dashboard shown in Figure 1, which operationalizes metacognitive reflection through post-session visual analytics.
- Ethical inclusion: grounded in Nussbaum’s capabilities approach [64], which emphasizes justice, empathy, and the dignified recognition of each learner’s agency, and informed by the ethics of care perspective that situates teaching as a relational and moral practice [36].

To strengthen conceptual clarity, Table 4 provides an operational glossary of the four core competencies cultivated within the E-MOTE framework. Each construct is defined according to its theoretical grounding and linked to observable indicators relevant to teacher-training contexts.

Conceptually developed through iterative engagement with the AI–FACS–VR pipeline, these competencies align with evidence that SEL programs improve classroom climate and outcomes [65]. Beyond individual competencies, E-MOTE emphasizes a broader ethical stance toward the design and adoption of educational technologies. Key challenges related to bias, privacy, consent, and implementation costs are examined in the Limitations section. Zawacki-Richter et al. [66] observed that excluding teachers from AI system design creates a risk of pedagogical misalignment. Subsequent literature confirms this risk, noting consequences such as mistrust, poorly aligned tools, and missed educational objectives.

In response, human-centered AI principles underscore participatory design approaches that emphasize safety, reliability, and trustworthiness [67]. This vision reinforces the role of teachers as empowered decision-makers who interpret, rather than passively accept, AI-generated feedback. In E-MOTE, teachers remain central agents who actively co-construct ethical, inclusive, and perceptually aware classroom practices.

Collectively, these competencies demonstrate how E-MOTE extends traditional teacher-training paradigms by linking micro-expressive attunement with ethical and reflective dimensions. Through the iterative AI–FACS–VR pipeline, the framework aims to transform emotional insight into pedagogically actionable professional awareness, embedding ethical awareness and inclusivity at the core of professional growth.

## 5. Experimental Validation Strategy

E-MOTE is a conceptual framework grounded in interdisciplinary research across neuroscience, education, and affective computing, deliberately designed as a preparatory and structuring phase for future empirical implementation rather than as a fully deployed system. Although prior research has demonstrated the benefits of emotionally attuned teaching [2], these findings have not yet been examined within the specific AI–FACS–VR configuration proposed by E-MOTE. Accordingly, this section outlines a concise, three-phase validation roadmap to guide future empirical work while clearly distinguishing the present conceptual contribution from its forthcoming validation.

Phase 1—Technical validation and feasibility.

This phase focuses on assessing the technical robustness and basic usability of the AI–FACS–VR module in controlled settings. Core objectives include evaluating the accuracy of FACS-based AU decoding applied to virtual student avatars, examining system responsiveness and usability, and initiating bias auditing procedures. Particular attention is devoted to mitigating demographic and representational bias through culturally diverse, FACS-annotated datasets to prevent systematic misclassification of affective cues [61].

Phase 2—Pedagogical validation and short-term efficacy.

The second phase investigates the framework’s impact on teacher learning within structured training contexts. Emphasis is placed on assessing improvements in teachers’ noticing and interpretation of micro-expressions, alongside teachers’ perceived pedagogical utility, ethical acceptability, and professional alignment. This phase also initiates cross-cultural calibration and validation, examining how cultural norms shape AU interpretation and feedback design, in line with international competence frameworks such as the OECD PISA Global Competence Framework [68].

Phase 3—Ecological and longitudinal validation.

The final phase addresses transfer to practice and longer-term impact. It examines whether perceptual competencies developed through E-MOTE-supported training translate into more responsive and inclusive classroom behaviors, as well as potential downstream effects on classroom climate and student well-being. This phase also considers scalability and implementation requirements, including institutional adoption and sustainability, under established ethical governance principles for AI in education [20].

Across all phases, validation will rely on a coherent combination of technical, pedagogical, and reflective indicators, including AU recognition accuracy, usability measures, perceived ethical acceptability, and observational indicators of emotional responsiveness. Detailed experimental protocols, sampling strategies, and measurement instruments are intentionally deferred to subsequent empirical publications dedicated specifically to validation.

## 6. Discussion

This section examines the theoretical and practical implications of the E-MOTE framework, underscoring its potential contributions to both teacher training and inclusive pedagogy. The analysis builds on existing scholarship in affective computing and simulation-based instruction, while addressing the conceptual and methodological gaps that E-MOTE seeks to bridge.

Moving from the micro-level of individual classroom practice to the broader landscape of educational systems, this section explores the prospective applications of E-MOTE, with particular reference to established simulation-based models such as SimSchool. Although E-MOTE and SimSchool both rely on simulation as a pedagogical medium, the proposed framework is positioned as a conceptual evolution in terms of pedagogical focus, methodological integration, and ethical design. SimSchool, widely used in teacher education programs, provides decision-based simulations designed to enhance teachers’ cognitive understanding of classroom dynamics and learner variability [16,17]. However, its primary emphasis remains on macro-level instructional decisions within scripted scenarios, and current descriptions do not report the integration of real-time affective inference or validated micro-expression decoding.

As illustrated in Table 5, E-MOTE builds on the established theoretical foundations of simulation-based training but seeks to extend them by addressing a relatively under-explored dimension of teacher competence: the real-time perception and interpretation of subtle, non-verbal emotional cues.

As no empirical testing has been conducted yet (see Section 5), the considerations that follow are presented as theoretically grounded hypotheses rather than as evidence of pedagogical effectiveness. Drawing on the internal logic of the E-MOTE framework and on established research on teachers’ social and emotional competence, the model is conceptually aligned with approaches that emphasize emotional attunement as a foundational professional resource in teaching practice [2,8].

Within this theoretical perspective, E-MOTE articulates a coherent mechanism through which emotional perception, awareness, and regulation may be progressively cultivated through guided and reflective practice in immersive environments. Insights from social and emotional learning research indicate that teachers’ emotional attunement is theorized to be associated with classroom climate and pedagogical responsiveness [65]. Consistent with this literature, the implications discussed here should be interpreted exclusively as theoretically informed assumptions intended to guide future empirical validation, rather than as claims of realized pedagogical impact.

The application prospects of the framework can be articulated across three key domains:

- Initial teacher training: universities could embed E-MOTE modules to provide foundational training in emotional competence, combining theoretical instruction on FACS with immersive VR simulations to cultivate perceptual skills from the outset of a teaching career.
- Ongoing professional development: for in-service teachers, E-MOTE could offer advanced modules tailored to specific challenges, such as managing inclusive classrooms or recognizing cross-cultural emotional expressions, supported by high-fidelity VR simulations for deliberate practice.
- Reflective inquiry tool: post-simulation dashboards are envisioned as providing structured indicators of teachers’ “noticing” skills, making E-MOTE a potential resource for self-assessment and coaching within professional learning communities.

Looking ahead, successful implementation will require careful attention to teacher readiness and cognitive load. Minimizing overload involves not only technical refinements but also alignment with evidence-based guidelines on the interplay between emotion, cognition, and learning as identified in contemporary neuroscience [69].

The technical–ethical constraints arising from FACS-driven biometric rendering, particularly when minors are involved, do not function as implementation barriers but as structural design conditions shaping VR avatar fidelity, re-identification risk auditing, data minimization logic, and scalable adoption.

Overall, E-MOTE has the potential to evolve from a conceptual framework into a systemic innovation in teacher education—provided its deployment remains participatory, ethically grounded, and empirically validated. Its distinctive contribution lies in the proposed integration of FACS-informed micro-expression analysis, AI-driven feedback, and immersive VR within a unified training ecosystem. This evolution would represent a paradigm shift from behavior-oriented simulation to perception-oriented professional learning.

A key priority is the active involvement of learners as stakeholders. Systematically incorporating student perspectives into the design and evaluation of educational interventions not only enhances effectiveness but also fosters a more inclusive and participatory learning culture. Integrating student voice reinforces pedagogical robustness by ensuring that teacher education remains attuned to evolving classroom needs and lived experiences [70].

## 7. Limitations

While E-MOTE proposes an integrated and conceptually robust framework for emotion-aware teacher training, several limitations must be acknowledged, including the absence of current empirical validation. Second, its reliance on AI-driven facial analytics introduces potential risks of algorithmic bias and cultural misclassification, which necessitate carefully designed ethical safeguards and the use of culturally diverse datasets [71,72]. Moreover, practical challenges related to teacher readiness, technostress, and VR-related discomfort may constrain its real-world applicability. Additionally, the acquisition and use of facial data required for high-fidelity avatar animation introduces further constraints. To minimize data exposure and prevent the transmission of biometric information to external providers, future implementations will prioritize locally deployed AI models [73]. While this approach enhances data protection, it may also reduce model accuracy due to the limited computational resources typically available in institutions with constrained budgets. Moreover, the storage and governance of the facial landmark data needed for realistic FACS-based animation require stringent safeguards, particularly when such data are derived from minors. Ensuring secure handling, limited retention, and ethically compliant processing of these sensitive datasets will therefore be essential for the responsible development of the system [74]. Furthermore, when biometric facial data derive from minors, CAD-to-FACS fidelity benchmarking must be governed through formalized consent pipelines, re-identification risk auditing, and institutionally supervised storage procedures, preventing dataset circulation across non-accredited providers.

Specific pedagogical and institutional constraints include: (i) difficulties in the integration with established teaching models. Teachers may need to adapt lesson plans to accommodate the technology, potentially diluting core pedagogical principles if not carefully managed. (ii) Traditional assessment methods (quizzes, written reflections) may not capture the nuanced affective data that E-MOTE provides. (iii) Effective use of the platform requires teachers to understand both the technical operation of the VR environment and the interpretation of affective data. Without comprehensive professional development, teachers may experience cognitive overload, leading to superficial or incorrect application of the system. (iv) The presence of real-time emotion monitoring could inadvertently influence learner behavior, reducing autonomy or fostering performance anxiety. Balancing supportive feedback with respect for individual learning styles is essential to avoid unintended negative impacts on motivation.

These limitations collectively underscore the need for iterative validation, contextual sensitivity, and adaptive implementation strategies.

To address these challenges, the research agenda should run in parallel with sustained efforts in three key areas:

- Data privacy and governance: ensure granular informed consent, data minimization, and robust pseudonymization protocols throughout all phases, subject to independent ethical oversight bodies.
- Teacher readiness and technostress: identify, design, and test effective support structures, such as structured professional development pathways and technical coaching interventions.
- Health and safety in VR environments: empirically validate safety protocols (session durations, debriefing practices) to reduce risks of simulator sickness and visual fatigue [71].

Future implementations should also account for infrastructural disparities across educational settings, as access to VR equipment and AI-based analytics may vary significantly between institutions, particularly in under-resourced or geographically peripheral contexts.

In practical terms, this agenda may benefit from multi-stakeholder funding partnerships (Erasmus+, Horizon Europe) and strong transparency commitments. Future pilot studies should be designed in accordance with reproducible research principles, including public documentation of AI–FACS–VR design parameters, data governance standards, and usability assessment protocols, in alignment with ethical AI guidelines and open science best practices.

While the proposed phased validation plan outlines a pathway toward methodological rigor and ethical alignment, empirical implementation remains essential to determine whether the conceptual strengths of E-MOTE may yield measurable improvements in classroom responsiveness, emotional competence, and student outcomes. These limitations and dependencies set the stage for future developments and final reflections on the broader trajectory of the framework.

Importantly, although the proposed model harnesses affective computing, the framework is explicitly designed to ensure that human oversight remains central to all interpretive and pedagogical decisions. In line with current ethical guidelines for trustworthy AI in education, emotion-sensitive analytics are positioned not as replacements for professional judgment, but as tools to inform reflective practice and foster teacher agency. Sustained attention to algorithmic transparency and human–machine complementarities will thus be essential throughout future implementation phases.

## 8. Conclusions

This paper proposes E-MOTE (Emotion-aware Teacher Education Framework), an ethically grounded conceptual model aimed at enhancing teacher education through the integrated use of the Facial Action Coding System (FACS), Artificial Intelligence (AI), and Virtual Reality (VR). Anchored in the ability model of emotional intelligence [6] and informed by contemporary research on affective computing and immersive learning, E-MOTE offers a coherent and ethically grounded blueprint for developing teachers’ perceptual and emotion-aware competencies.

A staged validation plan has been outlined to examine the technical reliability, pedagogical relevance, and cultural responsiveness of E-MOTE, in alignment with design-based research principles [15] and current calls for human-centered and trustworthy AI in education [20,26].

This conceptual multilayered scaffolding should be interpreted as a foundation for transparent, reproducible, and ethically bounded empirical implementations, ensuring that future iterations remain governed by fairness-auditing and human oversight commitments. Accordingly, and consistent with the design-only scope of the present proposal, E-MOTE does not permit any generalization regarding learning outcomes prior to empirical validation. Future empirical studies will assess its effectiveness in authentic classroom contexts, particularly in addressing documented limitations in teachers’ accuracy when judging students’ subjective well-being [75]. At present, E-MOTE offers researchers and practitioners with a structured, transparent, and theoretically robust model for next-generation simulation-based teacher training.

By articulating a transparent, ethically governed and theoretically grounded ecosystem for emotion-aware teacher training, E-MOTE offers a concrete conceptual scaffold upon which future empirical, technological and pedagogical developments can be systematically constructed.

## Figures and Tables

**Table 1:** Terminological clarification within the E-MOTE framework.

Level	Definition	Domain	Pedagogical Focus
Emotion detection	Computational identification of facial Action Units (AUs) and affective cues processed by AI.	Technological (AI–FACS)	Data acquisition and emotion inference.
Emotion perception	Teacher’s immediate recognition of learners’ emotional signals in context.	Human- perceptual	Observing and interpreting emotional cues.
Emotion awareness	Metacognitive understanding of perceived emotions and their implications.	Reflective- metacognitive	Reflecting on emotional dynamics in interaction.
Emotion regulation	Adaptive adjustment of communication and teaching strategies in response to emotions.	Behavioral-pedagogical	Modulating tone, pace, and feedback to maintain engagement.

**Table 2:** Mapping of key design features in selected AI- and VR-based approaches to teacher training (2022–2024).

Study	FACS Decoding	AI Real-Time Feedback	VR Immersion	Pedagogical Integration	Ethical Safeguards
SimSchool [16]	✘	✔	✘	✘	✘
TeachLivE [31]	✘	✘	✔	Partial	✘
ClassSim [32]	✘	✘	✔	✔	✘
E-MOTE	✔	✔	✔	✔	✔

E-MOTE is included for contextual positioning only and represents a proposed framework, not an empirically validated system. Legend. ✔ = explicitly designed or described; ✘ = not addressed, Partial = limited, exploratory, or insufficiently detailed. For proposed frameworks (E-MOTE), ✔ indicates conceptual design intent rather than empirical implementation.

**Table 3:** Pedagogically relevant facial action units (AUs) and their implications.

AU Code & Name	Muscle Movement	Possible Affective Signals	Potential Pedagogical Significance & Response
AU1: inner brow raiser	Eyebrows raised inward	Indicators potentially associated with worry, sadness, or cognitive effort	May indicate a student is struggling with a concept. A teacher could respond with a clarifying question, offer encouragement, or check for understanding
AU4: brow lowerer	Eyebrows lowered/drawn together	Patterns commonly associated with frustration, anger, or high cognitive load	Signals rising frustration or cognitive load. This is a key moment to intervene with scaffolding, a short break, or by re-framing the task to prevent disengagement
AU5: upper lid raiser	Upper eyelid raised, eye widening	Signals often linked to surprise, vigilance, or heightened alertness	Pedagogical cue: monitor for anxiety or startle response; slow pace or provide reassurance

**Table 4:** Core professional competencies cultivated through the E-MOTE framework, with corresponding theoretical references and operational indicators for teacher education.

Competence	Theoretical Foundation	Operational Indicators in Teacher Training
Emotional attunement	Based on affective neuroscience and interpersonal neurobiology; aligned with SEL frameworks.	Accurate recognition of facial micro-expressions (AUs), appropriate modulation of emotional tone, and synchrony with students’ affective cues during immersive simulations.
Empathic responsiveness	Draws on empathy theory and the Prosocial Classroom model.	Demonstrates context-sensitive reactions, verbal and non-verbal mirroring, and adaptive feedback to students’ emotions.
Reflective growth	Informed by reflective practice and transformative learning.	Uses post-session dashboards to identify strengths and areas for improvement; integrates emotional feedback into ongoing professional reflection.
Ethical inclusion	Grounded in UNESCO AI-ethics principles and culturally responsive pedagogy.	Applies fairness-aware strategies, ensures representation of diverse emotional expressions, and reflects critically on bias and ethical challenges within simulated interactions.

**Table 5:** Comparative conceptual positioning of SimSchool and the proposed E-MOTE framework.

Dimension	SimSchool	E-MOTE
Primary training focus	Classroom management and instructional decision-making using AI-driven learner profiles	Perceptual acuity and emotional responsiveness, conceptually supported by FACS-informed micro-expression analysis, immersive VR, and AI-driven adaptive feedback
Underlying approach	Relies on scripted learner behaviors and pre-programmed scenarios, not real affective data	Conceptual use of FACS-based emotional analytics to interpret non-verbal affective cues within simulated interactions, combined with competency-oriented training pathways
Feedback mechanism	Primarily post-simulation analysis of teacher actions on student macro-behaviors (e.g., “engagement” score)	Proposed real-time and post-session feedback on perceptual performance (highlighting potential cues of frustration, such as AU4, in a given student avatar), provided both in-VR and via post-simulation analytics
Technological components	AI-driven behavioral simulation of students	Conceptually integrated ecosystem including FACS, AI analytics, VR immersion, multimodal feedback, and culturally responsive calibration
Scope of innovation	A mature platform for simulating classroom dynamics and pedagogical strategies	Conceptually advances the field by targeting the foundational skill of emotion perception, uniting FACS-informed micro-expression analysis, immersive practice, and adaptive feedback within a single training ecosystem designed for scalability

## Data Availability

No new data were created or analyzed in this study. Data sharing is not applicable to this article.
